# Minipterional craniotomy for resection of epileptogenic cavernous malformation

**DOI:** 10.3171/2024.4.FOCVID2441

**Published:** 2024-07-01

**Authors:** Alexander Chartrain, Carli Bullis, Nicholas Sader, Peter A. Chiarelli, Madeline Kahan, Brittany Jordan, Latanya Agurs, Michele Van Hirtum-Das, Asri Yuliati, Sucheta Joshi, Jason Chu

**Affiliations:** Departments of 1 Neurosurgery and; 4 Neurology and Pediatrics, Keck School of Medicine of USC, Los Angeles, California;; 2 Hawaii Pacific Health, Honolulu, Hawaii;; Divisions of 3 Neurosurgery and; 5 Neurology, Children’s Hospital Los Angeles, California

**Keywords:** epilepsy, cavernous malformation, pediatric

## Abstract

Epilepsy is a common symptom of pediatric cavernous malformations. In medically refractory patients, surgery can achieve high seizure freedom rates with low morbidity. This video depicts the use of a minipterional craniotomy and transsulcal resection of a frontal opercular cavernous malformation in a 13-year-old female with medically intractable epilepsy. At 1-year follow-up, she was evaluated as Engel class I with a significant improvement in her quality of life. Principles of cavernous malformation resection for the treatment of epilepsy are also reviewed.

The video can be found here: https://stream.cadmore.media/r10.3171/2024.4.FOCVID2441

## Transcript

We present a case of a pediatric patient with medically refractory epilepsy due to an epileptogenic cavernous malformation. Surgical resection was completed through a minipterional craniotomy and transsulcal approach to the lesion.

### 0:34 Case Presentation.

The patient is a 13-year-old female that presented to our Comprehensive Epilepsy Center with 2-year history of seizures. At time of evaluation, she was having focal aware seizures with a semiology of her body feeling numb and tingling, behavioral arrest, left-hand dystonic posturing, and facial twitching, all without loss of consciousness. Although these events were brief, her seizures would occur 10–12 times a day, including waking her up at night. She was on 3 antiseizure medications; however, her seizures remained poorly controlled.

She had no significant birth or family history, including a familial history of cavernous malformations. She had normal development and met all her milestones. She had no history of any epilepsy risk factors. She had a normal neurological exam. Socially, her epilepsy had a significant impact on her life—she was unable to achieve a full night of sleep. At school, she experienced decreased focus and concentration with a decline in her academic performance. She stopped playing soccer. She also experienced decreased energy, mood changes, and depression.

### 1:34 Imaging.

An MRI scan demonstrated a single cavernous malformation within the right pars opercularis and located at the depth of a sulcus. On this T2-weighted image, the classic popcorn-appearing lesion is visualized with a rim of hemosiderin. There was no previous history of hemorrhage. A developmental venous anomaly was not well appreciated.

### 1:55 Epilepsy Surgery Workup.

She underwent a surgical workup at our center. To summarize, her phase I captured numerous ictal events localized to her right frontal area. Neuropsychological testing was suggestive of frontal lobe dysfunction, although it was not strongly lateralizing. An fMRI demonstrated the lesion was in a noneloquent area with left-sided language and her motor area in a safe distance away from the lesion.

With concordant clinical, electrophysiological, and imaging data, she was recommended to undergo image-guided surgical resection of her cavernous malformation with intraoperative electrocorticography. We elected to approach the cavernous malformation through a minipterional approach that would allow for exposure of the frontal operculum. Compared to a standard pterional craniotomy, this approach minimizes temporalis trauma and atrophy, decreases the risk of frontalis palsy or inadvertent injury to the superficial temporal artery, and has excellent cosmetic outcomes.^1^

The cavernous malformation was located at the depth of a sulcus and approached via a transsulcal route. On the right, the contrasted T1-weighted image has been rotated into the operative view to better appreciate the surface landmarks and surgical anatomy. The blue arrow depicts the sulcus overlying the cavernous malformation.

Here is the correlation between her preoperative imaging and intraoperative anatomy.

### 3:13 Surgical Resection.

The arachnoid overlying the sulcus is opened widely. There are several techniques for arachnoid dissection, and the jeweler forceps are useful instruments to gain initial access by elevating and gently spreading this layer. Once opened, the sulcus can be identified and dissected open with the tips of the bipolar forceps. Deep arachnoid adhesions can be divided to facilitate superficial dissection. Adequate exposure along the entire length of the sulcus is required for visualization of the depth of the sulcus. During the dissection, pial bleeding can be controlled with a small piece of Surgicel and tamponade.

The sulcus is opened with the bipolar oriented perpendicularly and gentle pressure to reach the depth of the sulcus. The superficial-most aspect of the cavernous malformation can be seen. Lesionectomy begins at the interface between the cavernous malformation and surrounding gliotic white matter. The interface is cauterized and cleared with the suction in a circumferential manner around the cavernous malformation. Here, we are starting our dissection along the posterior aspects of the lesion.

The microscope has been rotated to facilitate dissection along the anterior and inferior portions of the lesion. Once the cavernous malformation is completely free from the surrounding white matter, it can be removed en bloc. Of note, no developmental venous anomaly was appreciated in the vicinity of the cavernous malformation, but these should be preserved if identified.

There is data to suggest removal of the surrounding hemosiderin rim improves postoperative seizure freedom rates. These areas can be identified as "yellowish" areas and have a different consistency compared to normal white matter. Here we’re using the bipolar and suction; however, ultrasonic aspiration is an alternative tool for efficient resection of the hemosiderin rim. Hemostasis is achieved and the cavity is reinspected for any remnant areas of hemosiderin. Here is our final view from our transsulcal approach to this lesion prior to closure.

### 5:53 Postoperative Course.

Intraoperative ECoG showed significant improvement after resection. Postoperatively she was neurologically intact and, importantly, had immediate cessation of her seizures. Postoperative MRI demonstrated gross-total resection of the cavernous malformation and hemosiderin rim. She was discharged on postoperative day number 3.

### 5:12 Long-Term Follow-Up.

At her 1-year postoperative visit, she was Engel I and seizure free. At her last neurosurgical follow-up, 3 years postoperatively, she had excellent control of her epilepsy. Her 6-month postoperative EEG showed frontocentral slowing consistent with her previous surgery and no epileptiform discharges. Postoperative MRIs have been reassuring. Importantly, with her epilepsy under control, she had significant improvements in her mood, social interactions, school performance, and overall quality of life.

### 6:42 Key Points.

Thirty percent of pediatric patients with cavernous malformations may develop medically refectory epilepsy. Surgical resection of concordant, epileptogenic lesions can result in epilepsy control rates between 80% and 90%. Early surgical intervention may result in superior postoperative seizure freedom rates, and surgical resection should include hemosiderin rim to maximize seizure outcomes. Although not utilized in this case, there is early data to support laser interstitial thermal therapy (LITT) as an alternative treatment option for epileptogenic cavernous malformation.

### 7:15 References.

See papers for more details.^1–6^

## Disclosures

The authors report no conflict of interest concerning the materials or methods used in this study or the findings specified in this publication.

## Author Contributions

Primary surgeon: Chu. Assistant surgeon: Chartrain, Bullis. Editing and drafting the video and abstract: Chu, Chartrain, Sader. Critically revising the work: Chu, Chartrain, Sader, Chiarelli, Joshi. Reviewed submitted version of the work: Chu, Chartrain, Bullis, Sader, Chiarelli, Kahan, Jordan, Agurs, Yuliati, Joshi. Approved the final version of the work on behalf of all authors: Chu. Supervision: Chu. Neurophysiologic data: Van Hirtum-Das.

## Supplemental Information

### Patient Informed Consent

The necessary patient informed consent was obtained in this study.
